# Can medication-related osteonecrosis of the jaw be attributed to specific microorganisms through oral microbiota analyses? A preliminary study

**DOI:** 10.1186/s12903-024-03945-z

**Published:** 2024-02-01

**Authors:** Heon-Young Kim, Young-Soo Jung, Wonse Park, Yoon Jeong Choi, Jun-Young Kim

**Affiliations:** 1 Department of Oral and Maxillofacial Surgery, Ewha Womans University Medical Centre, Seoul, Republic of Korea; 2 Department of Oral and Maxillofacial Surgery, Yonsei University College of Dentistry, 50-1 Yonsei-ro, Seodaemun-gu, Seoul, 03722 Republic of Korea; 3 Department of Advanced General Dentistry, Yonsei University College of Dentistry, Seoul, Republic of Korea; 4 Institute for Innovation in Digital Healthcare, Yonsei University, Seoul, Republic of Korea; 5 Department of Orthodontics, The Institute of Craniofacial Deformity, Yonsei University College of Dentistry, Seoul, Republic of Korea

**Keywords:** Medication-related osteonecrosis of the jaw, Microbiome, Oral bacteria, Metagenomics

## Abstract

**Background:**

Medication-related osteonecrosis of the jaw (MRONJ) can cause significant pain and loss of aesthetics and function if not treated properly. However, diagnosis still relies on detailed intraoral examinations and imaging. Prognosis varies even among patients with similar stages or conditions of MRONJ, emphasizing the need for a deeper understanding of its complex mechanisms. Thus, this study aimed to identify the oral microbiota of patients with MRONJ.

**Methods:**

This single-center prospective cohort study included patients with confirmed MRONJ who visited the Department of Oral and Maxillofacial Surgery at Yonsei University Dental Hospital between 2021 and 2022. Oral swab samples were collected from the affected and unaffected sides of each patient. The composition and enumeration of the microbial communities were analyzed, and the diversity was compared to verify ecological changes in the groups using a next-generation sequencing-based 16S metagenomic analysis. A statistical analysis was performed using Wilcoxon signed-rank test with SPSS version 22, and values of P less than 0.05 were considered statistically significant.

**Results:**

The final study sample included 12 patients. The mean age was 82.67 ± 5.73 (range, 72–90) years. Changes in microbial composition were observed at different taxonomic levels (phylum, genus, and species). The identified microorganisms were commonly associated with periodontitis, gingival disease, and endodontic infection, suggesting a multifactorial etiology of MRONJ.

**Conclusions:**

Although this study is based on a small number of cases, it shows that MRONJ is not caused by a specific microorganism but can rather be caused by a variety of factors. By addressing these findings in large-scale studies, the significance of oral microbiome in pathogenesis can be further elucidated and can facilitate the development of effective therapeutic interventions for patients with MRONJ.

**Supplementary Information:**

The online version contains supplementary material available at 10.1186/s12903-024-03945-z.

## Introduction

Medication-related osteonecrosis of the jaw (MRONJ) is an uncommon condition that may occur following exposure to antiangiogenic and antiresorptive agents [1]. The term MRONJ was first introduced in 2003 [2–5]. It is one of the most severe complications reported in the last two decades from the use of the aforementioned agents. Most cases of MRONJ present as exposed bone in the maxillofacial region, although cases of unexposed MRONJ have also been reported [6–9]. According to current information, MRONJ can be caused by various drugs, including oral or intravenous bisphosphonates, receptor activator of nuclear factor κB ligand inhibitors, and monoclonal antibodies. Numerous clinical and pharmacological studies have demonstrated that these drugs are effective when used for several bone disorders, including reducing fracture incidences by increasing bone density and preventing bone metastases from malignancies [10, 11]. However, as the number of patients receiving these drugs increases, the prevalence of MRONJ is also increasing [3, 12]. In the literature, the incidence of MRONJ has been reported to be 0.4–21%, depending on the drug administration route, dosage, and type [12–16]. MRONJ can cause significant pain as well as and loss of aesthetics and function if not treated properly. However, its diagnosis still relies on detailed intraoral examinations and imaging (orthopantomography and cone beam computed tomography). Prognosis varies even among patients with similar stages or conditions of MRONJ emphasizing the need for a deeper understanding of its complex mechanisms.

The pathogenesis of MRONJ remains unclear. Although various etiological markers have been suggested, they remain controversial. As such, there has been a lack of clear models to explain MRONJ, and the factors involved in its pathogenesis have only been hypothesized. Among these, it has been suggested that oral bacteria found in the bones may play a vital role in MRONJ pathophysiology [17] and reports that 82.18% of *Actinomyces* were detected in the infected bones of patients with MRONJ support an infectious etiology [18, 19]. Furthermore, although healthy maxillary and mandibular tissues are known to be resistant to oral bacterial flora, patients taking antiresorptive or antiangiogenic agents are vulnerable to bone infections and may develop MRONJ due to opportunistic infections involving bacteria and other microbes [20]. Moreover, local infections can lower the natural pH of the alveolar bone. Similarly, inappropriate surgery or prosthetic treatment may disrupt homeostasis and lead to pH changes that delay soft tissue healing, potentially affecting MRONJ development [21–23]. Although microbes such as fungi, viruses, and bacteria have been detected in exposed bones via histological examination of clinical specimens [24–27], it is still unclear whether specific oral microbes are associated with MRONJ pathophysiology [28]. To elucidate the pathogenesis of MRONJ and its associated metabolic processes, it is necessary to identify the specific microbial species involved [17, 18, 29].

The human microbiome has become the background of ecological theory [30], and is known to have a crucial function in metabolic processes, nutrition, homeostasis, defense against harmful infections, and even genetic influences [31–33]. Therefore, it is expected that understanding the oral microbiome may help to identify etiology of MRONJ. Previous culture-based studies have limitations, leading to a growing interest in exploring the oral microbiome using next-generation sequencing (NGS) techniques.

Thus, this study aimed to identify the oral microbiome in patients with MRONJ using an NGS-based 16S metagenomic analysis. We hypothesized differences in microbial communities between affected and unaffected oral mucosa areas in these patients. By analyzing the composition and enumeration of these communities, and by comparing their diversity, we aim to understand the ecological change and their implications in MRONJ pathogenesis.

## Materials and methods

### Study sample

Patients diagnosed with MRONJ at the Department of Oral and Maxillofacial Surgery, Yonsei University Dental Hospital (2021–2022), were recruited for this study. After thoroughly explaining the study details, written consent was obtained from each participant. The study adhered to the Declaration of Helsinki and received approval from the Yonsei University Dental Hospital’s Institutional Review Board (IRB No. 2–2022-0001).

Individuals who underwent clinical examination upon visiting the hospital, met the 2022 American Association of Oral and Maxillofacial Surgeons (AAOMS) diagnostic criteria for MRONJ, and had never been treated surgically or with antibiotic therapy, were included. The AAOMS diagnostic criteria were as follows:

1. Current or previous treatment with antiresorptive therapy alone or in combination with immune modulators or antiangiogenic medications.
2. Exposed bone or bone that can be probed through an intraoral or extraoral fistula(e) in the maxillofacial region that has persisted for more than 8 weeks.
3. No history of radiation therapy or metastatic disease to the jaws.

Exclusion criteria were as follows:

1. Non-compliance with AAOMS criteria.
2. Declining participation or inability to understand the consent form.
3. Bilaterally affected jaw preventing contralateral sample collection.
4. Recent surgery or antibiotic therapy (within 6 months).
5. Systemic conditions potentially affecting bacterial distribution (e.g., bacteremia, endocarditis, autoimmune diseases).
6. Active cancer or cancer diagnosis within the last 3 years, and those with xerostomia, or reduced salivary gland function.
7. Oral disease like mucositis or gastrointestinal conditions like reflux esophagitis, potentially altering oral microbial community.

#### Data collection methods

**Fig. 1:**
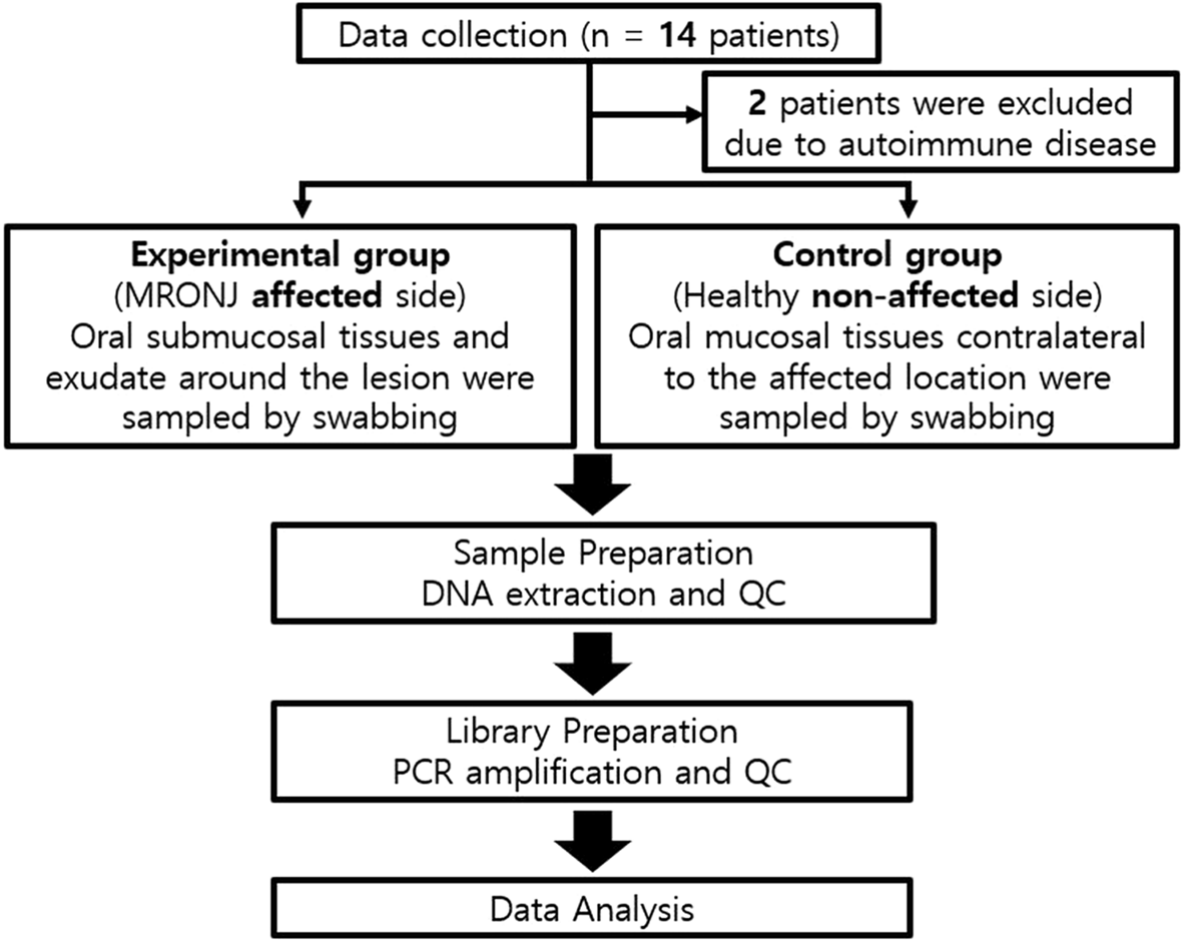
Study design flowchart. DNA, deoxyribonucleic acid; MRONJ, medication-related osteonecrosis of the jaw; PCR, polymerase chain reaction; QC, quality control

Figure 1 illustrates a schematic flowchart depicting the patient selection process. Data including age, sex, lesion location, underlying diseases, relevant medication history, and smoking/alcohol consumption status were collected through interviews and examinations. The MRONJ stage was determined by clinical examinations and diagnostic investigations, and radiologic examination confirmed the extent of the lesion.

Considering that the saliva secretion rate can affect the microbial environment of the oral cavity and varies among individuals and within the same individual depending on the situation and timing [34], samples were collected uniformly using the same method. Patients were advised not to drink, smoke, or take antibiotics 1 week before sample collection. They were also examined to ensure they had no oral or severe systemic diseases. On the day of sample collection, the patients were asked to refrain from eating and drinking for 1 hour before sampling.

**Fig. 2:**
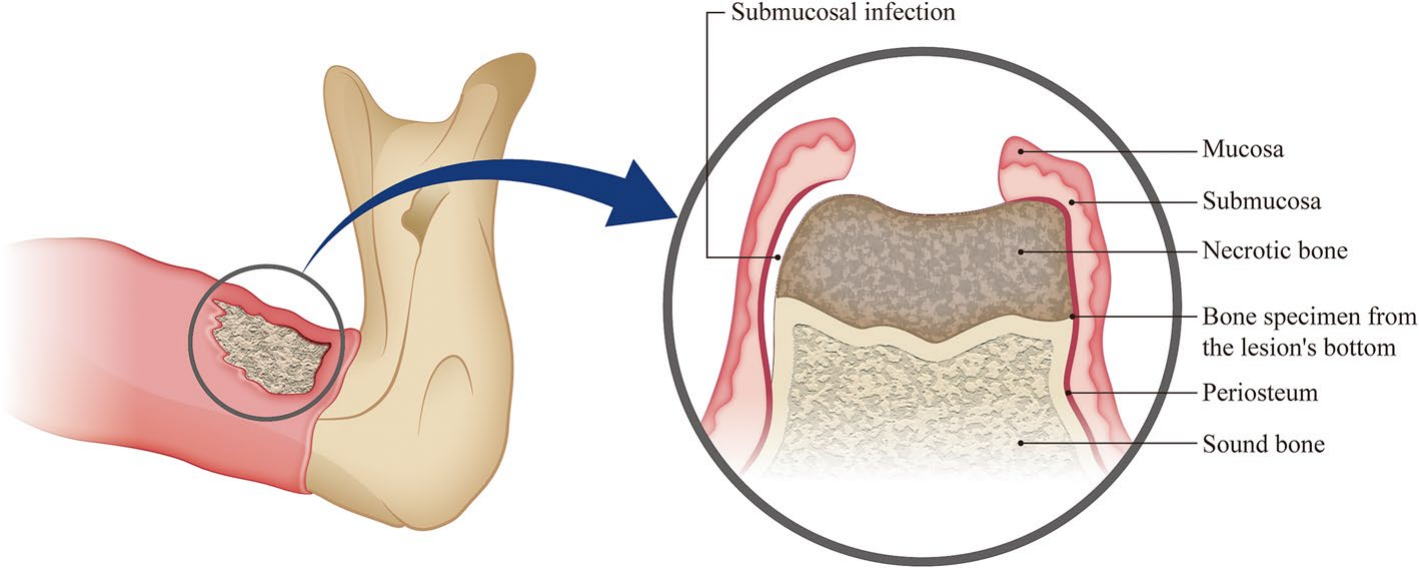
Schematic diagram of the affected area within the oral cavity. Samples were collected from the submucosal area

This study utilized a split-mouth design in which each patient served as both an experimental and control participant. The affected area of the jaw was targeted in the experimental group. Before any surgical intervention, the oral submucosal tissues and exudate around the lesion in the oral cavity were sampled using an OMNIgene OMR-110 kit (DNA Genotek Ottawa, Canada). First, saliva was removed by gently gargling with warm water and then air-dried to avoid disturbing the sampling site. Samples were obtained from the deepest part of the oral submucosal tissues and exudate around the lesion by swabbing for 30 seconds (Fig. 2). The unaffected contralateral jaw was included in the control group. Asymptomatic and normal oral mucosal tissues contralateral to the affected side were sampled in a similar manner. Sterile swabs were opened immediately before sample collection. Special care was taken to avoid contact with and contamination from other parts of the oral cavity. This sample collection procedure did not cause any pain or discomfort to the patients and was performed without local anesthesia. Clinical examinations and sampling of enrolled patients were performed only by Dr. JYK to reduce inter-investigator bias.

The collection protocol followed the oral sample collection procedure described in the Human Microbiome Project 1 [35]. The collected samples were assigned codes and anonymized before being stored in a refrigerator (− 20 to − 30 °C), with deoxyribonucleic acid (DNA) and ribonucleic acid (RNA) integrity maintained for 4 weeks at a normal room temperature range (21 to 27 °C). Samples were sent to the analysis institution within 2 weeks.

##### Sample preparation

The entire genomic DNA of the microorganisms collected from each sample was extracted using the DNeasy® PowerSoil® kit (Qiagen, Hilden, Germany), and the experiment was conducted according to the protocol of the DNA extraction kit. The samples were placed in a tube and homogenized with the solution for 10 minutes, and the homogenized supernatant was transferred to a 2 mL tube and centrifuged four times with the solution. The DNA extracted through this process was transferred to deionized water and stored at − 20 °C. The quality of the extracted microbial gDNA was examined using the equipment and stored at 4 °C for the next experiment. Quantification was then performed using Quant-IT PicoGreen (Invitrogen, Carlsbad, CA, USA).

##### Polymerase chain reaction amplification and sequencing

Sequencing of each 16S rRNA gene was performed according to Illumina 16S V3-V4 Metagenomic Sequencing Library protocols (Illumina, San Diego, CA, USA). This study targeted the V3–V4 hypervariable region of the 16S rRNA gene for metagenomic sequencing, which was performed according to the National Institutes of Health Human Microbiome Project protocol.

Polymerase chain reaction (PCR) amplification of the region was performed as follows: 2 ng of input gDNA was amplified with 5x reaction buffer, 1 mM dNTP mix, and 500 nM each of the universal F/R PCR primers and Herculase II fusion DNA polymerase (Agilent Technologies, Santa Clara, CA, USA). The cycle condition for the first PCR was 3 min at 95 °C for heat activation and 25 cycles of 30 sec at 95 °C, 30 sec at 55 °C, and 30 sec at 72 °C, followed by a 5-min final extension at 72 °C.

The universal primer pairs and Illumina adapter overhang sequences used for the first amplification were as follows: The initial PCR product was purified using AMPure beads (AgenCourt Biosciences, Beverly, MA, USA). Following purification, 2 μL of the first PCR product was amplified for final library construction using the Nextera XT Indexed Primer. The cycle conditions for the second PCR were the same as those for the first PCR, except for the 10 cycles. The PCR products were purified using AMPure beads. The final purified product was quantified using quantitative PCR according to Quantification Protocol Guide (KAPA Library Quantification kits for Illumina Sequencing platforms) and a TapeStation D1000 ScreenTape (Agilent Technologies, Waldbronn, Germany). Paired-end (2 × 300 bp) sequencing was performed by Macrogen using a MiSeq platform (Illumina, San Diego, CA, USA).

#### Data analysis

##### Microbiome sequencing analysis

After sequencing, the Illumina MiSeq Raw data were sorted by sample and paired-end FASTQ files were generated. The Cutadapt (v3.2) program was used in the pre-processing step to remove the sequencing adapter sequences and F/R primer sequences of the target gene region, followed by cutting the forward sequence (Read1) and reverse sequence (Read2) to 250 bp and 200 bp, respectively. The Divisive Amplicon Denoising Algorithm 2 (DADA2 v1.18.0) package in R (v4.0.3) was used to correct for errors in the amplicon sequencing process. Sequences with two or more expected errors were excluded from paired-end reads. After completing the pre-processing step, an error model was established for each batch of data and the noise for each sample was removed.

**Fig. 3:**
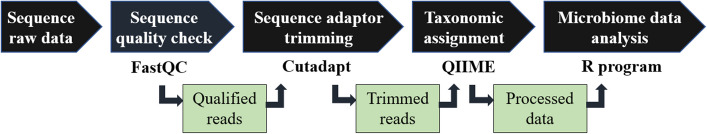
Workflow for analyzing the microbiome derived from the 16S rRNA. The programs specified were used for the listed steps of the microbiome analysis QC, quality control

After assembling error-corrected paired-end sequences into a single sequence, the DADA2 consensus method was used to remove chimera sequences and form amplicon sequence variants (ASVs). Additionally, to compare the microbial communities, the QIIME (v1.9) program was used to normalize the data by subsampling based on the read count of the sample with the minimum number of reads among all samples [36]. Each ASV sequence was subjected to a BLAST+ search against the reference database (DB) (NCBI 16S Microbial DB) to assign taxonomic information to the subject organism with the highest similarity. However, if the query coverage of the best hit matching the DB or the identity of the matched region was less than 85%, taxonomic information was not assigned. This workflow is illustrated in Fig. 3.

##### Statistical analysis

SPSS version 22 (IBM Corp., Armonk, NY, USA) was used for the statistical analyses of the clinical data, and a *P*-value of less than 0.05 was considered statistically significant. An operational taxonomic unit clustering analysis, a taxonomic profiling to identify specific bacteria, and alpha and beta diversity analyses were conducted. Data and statistical analysis throughout the experimental process were performed by Dr. HYK to minimize error.

## Results

### Study sample

A total of 14 patients were eligible, and 12 patients were eventually included.

### Participant characteristics

The participants’ characteristics are presented in Table 1. The mean age was 82.67 ± 5.73 (range, 72–90) years. Of the 12 subjects, 11 had osteoporosis and 1 had multiple myeloma. Regarding the MRONJ stage at the time of the hospital visit, 9 patients had stage II and three had stage III. The MRONJ site was the lower jaw in 9 patients and the upper jaw in three patients. The presumed trigger factors were tooth extraction in eight patients, periodontitis in three, and implantation in one. None of the 12 participants smoked or consumed alcohol. The microbiome profile of each participant was analyzed.

**Table 1:** Demographic and clinical information of the study patients

Patient	Age/Sex	Underlying Disease(s)	Drug Type and Duration	Stage/Location	Trigger Factor
**1**	85/F	Hypertension, osteoporosis	PO, risedronate 2017–2021	II/Mn Rt	Extraction
**2**	83/F	Hypertension, diabetes, osteoporosis	PO, ibandronate 2010–2020; SC, denosumab 2020–2021	II/Mn Rt	Extraction
**3**	81/F	Multiple myeloma	IV, zoledronic Acid 2020–2021	III/Mx Rt	Periodontitis
**4**	90/F	Osteoporosis	PO, ibandronate 2000–2010; IV, ibandronate 2011–2021	II/Mn Rt	Extraction
**5**	83/F	Hypertension, osteoporosis	PO, ibandronate 2016–2019; IV, ibandronate 2019 ~ 2021	II/Mx Rt	Extraction
**6**	80/F	Hypertension, diabetes, osteoporosis	PO, alendronate 2017–2022	II/Mn Rt	Implantation
**7**	88/F	Hypertension, osteoporosis	PO, alendronate 2016 ~ 2021	II/Mn Rt	Periodontitis
**8**	86/F	Hypertension, angina, osteoporosis	IV, pamidronate 2013–2022	II/Mn Lt	Extraction
**9**	87/F	Arrhythmia, osteoporosis	PO, ibandronate 2011–2013; PO, risedronate 2013–2018; PO, ibandronate 2019–2020; SC, denosumab 2021–2022	II/Mn Lt	Extraction
**10**	72/F	Osteoporosis	IV, zoledronate 2017–2021	II/Mn Lt	Extraction
**11**	72/F	Osteoporosis	SC, denosumab 2020–2022	III/Mx Lt	Periodontitis
**12**	85/F	Osteoporosis	IV, pamidronate 2017–2021	III/Mn Rt	Extraction

*F* female, *IV* intravenous, *Lt* left, *M* male, *Mn* mandible, *Mx* maxilla, *PO* per oral, *Rt* right, *SC* subcutaneous

### Microbial community diversity analysis

The taxonomic identification of the two groups is presented in Supplementary Table 1. The mean number of reads found in the collected samples was 92,638 ± 20,844 (range, 56,192–153,731). The mean number of reads used for analysis through filtering was 62,761 ± 16,817 (range, 29,510–97,604), and an ASV mean of 201 ± 98.2 (range, 85–549) was used for the analysis.

### Differences in bacterial phyla, genera, and species between groups (microbial taxonomy)

#### Oral microbiome analysis at the phylum level

**Fig. 4:**
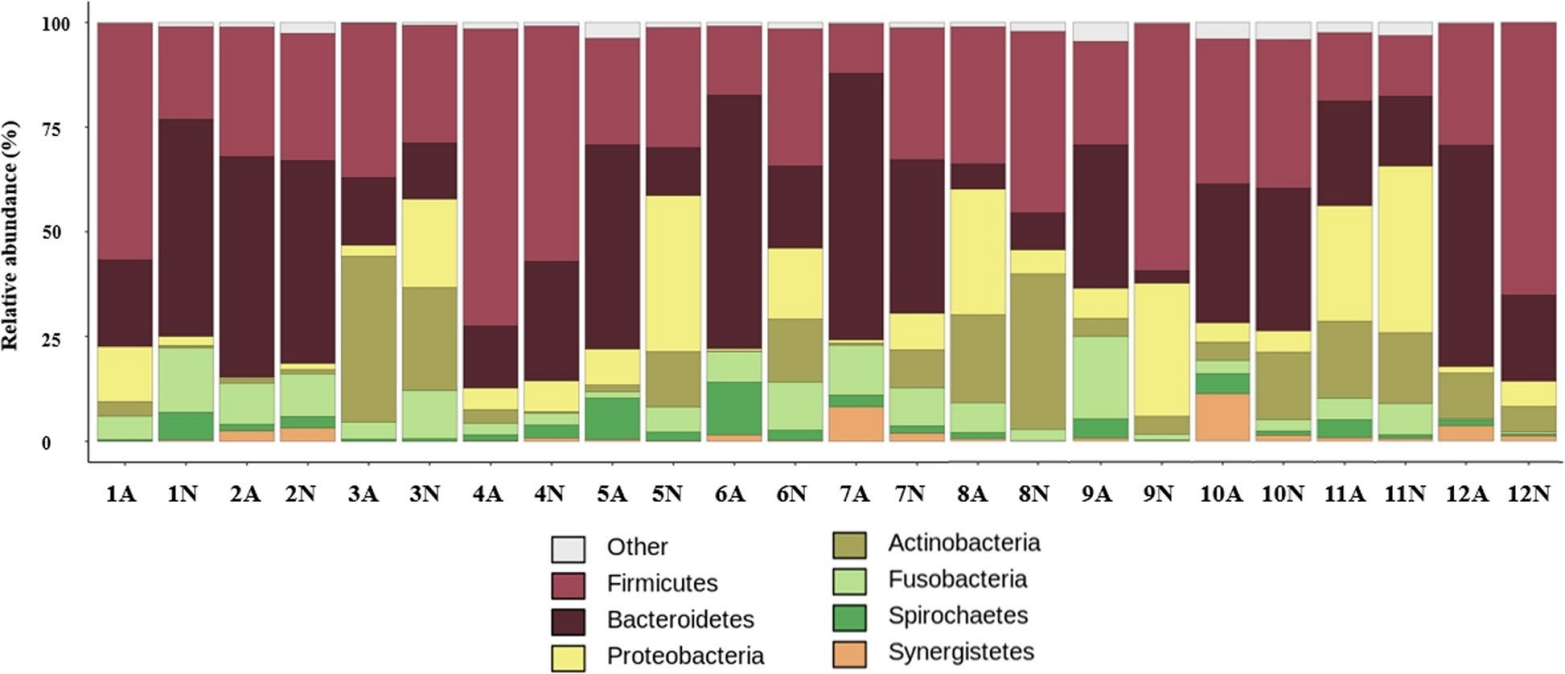
Oral microbiome analysis at the phylum level. A, affected group; N, unaffected group

The relative abundances between the two groups were compared to identify compositional differences in the oral microbiome (Table 2). A total of 18 were identified at the phylum level, with *Firmicutes*, *Bacteroidetes*, *Proteobacteria*, *Actinobacteria*, *Fusobacteria, Spirochaetes, and Synergistetes* accounting for over 90% (Fig. 4).

**Table 2:** Types and relative abundance of major microbiomes observed at the phylum level in both the groups

	Group	
Microbiome	Unaffected	Affected
***Firmicutes***	37.3%	32.2%
***Bacteroidetes***	24.4%	35.7%
***Proteobacteria***	15.3%	8.5%
***Actinobacteria***	12.0%	9.0%
***Fusobacteria***	6.7%	6.5%
***Spirochaetes***	1.8%	3.8%
***Synergistetes***	0.8%	2.5%
***Other***	1.7%	1.8%

**Fig. 5:**
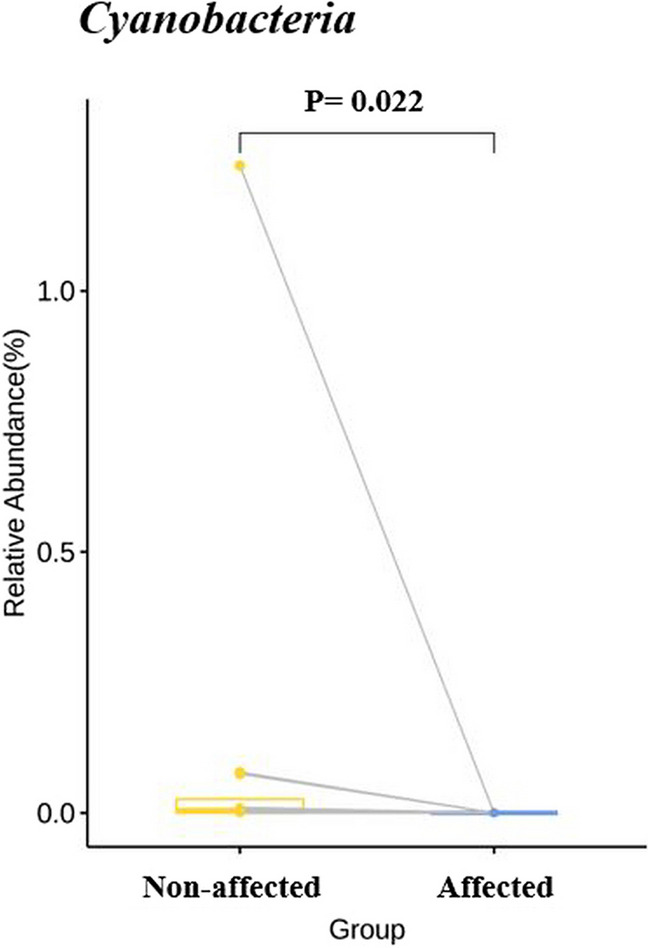
Oral microbiome comparison at the phylum level between the two groups. The yellow and blue boxes represent the unaffected and affected groups, respectively; the Y-axis shows their relative abundance as a percentage

Notably, *Cyanobacteria* had significantly higher abundance in the unaffected group than in the affected group. Although differences in other microbiome composition were observed between the two groups, these findings were not statistically significant (Fig. 5).

#### Oral microbiome analysis at the genus level

**Fig. 6:**
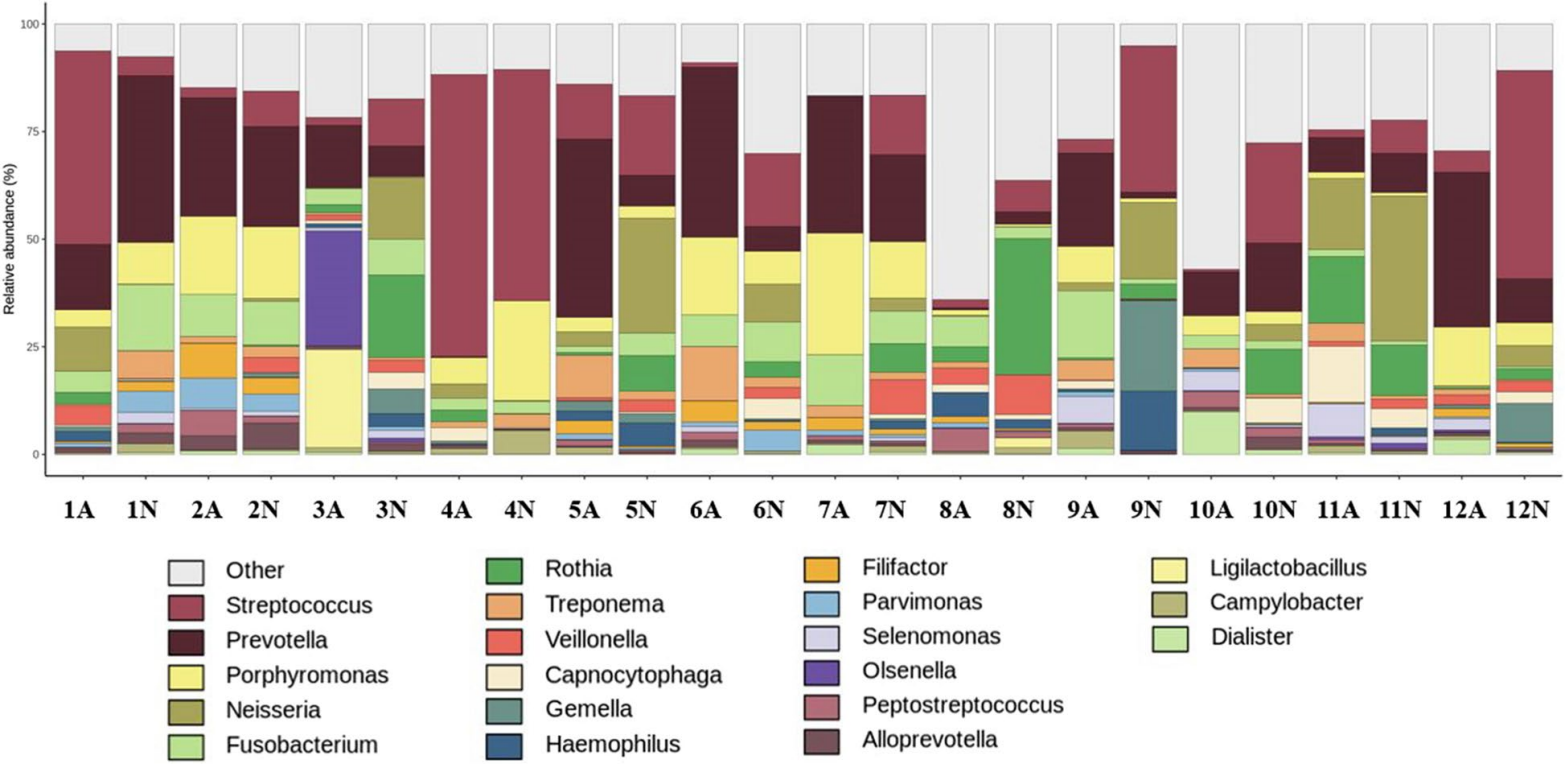
Oral microbiome analysis at the genus level. A, affected group; N, unaffected group

In total, 394 taxa were included in the genus analysis. Among these, 20 taxa were identified at the genus level (Table 3). Taxa with an average abundance value < 1% in the entire sample were labelled as “other” (Fig. 6).

**Table 3:** Type and relative abundance of major microbiomes observed at the genius level in both the groups

	Group	
Microbiome	Unaffected	Affected
Prevotella ^b^	11.8%	20.5%
Streptococcus ^b^	20.6%	11.8%
Porphyromonas ^b^	7.0%	8.9%
Fusobacterium ^b^	5.5%	5.8%
Neisseria^a^	9.5%	3.0%
Rothia	8.2%	2.3%
Veillonella^a^	2.8%	1.2%
Capnocytophaga	2.1%	1.8%
Treponema	1.8%	3.8%
Selenomonas	0.7%	2.0%
Campylobacter	1.0%	1.0%
Haemophilus	2.4%	0.9%
Parvimonas	1.4%	1.3%
Gemella	3.3%	0.4%
Alloprevotella	1.3%	1.0%
Filifactor	0.9%	1.9%
Peptostreptococcus	0.8%	1.8%
Olsenella	0.2%	2.4%
Ligilactobacillus	0.2%	1.9%
Dialister^a^	0.3%	1.7%
Other	18.2%	24.6%

^a^Significant difference between the two groups, ^b^abundant microbial taxa in both the groups

**Fig. 7:**
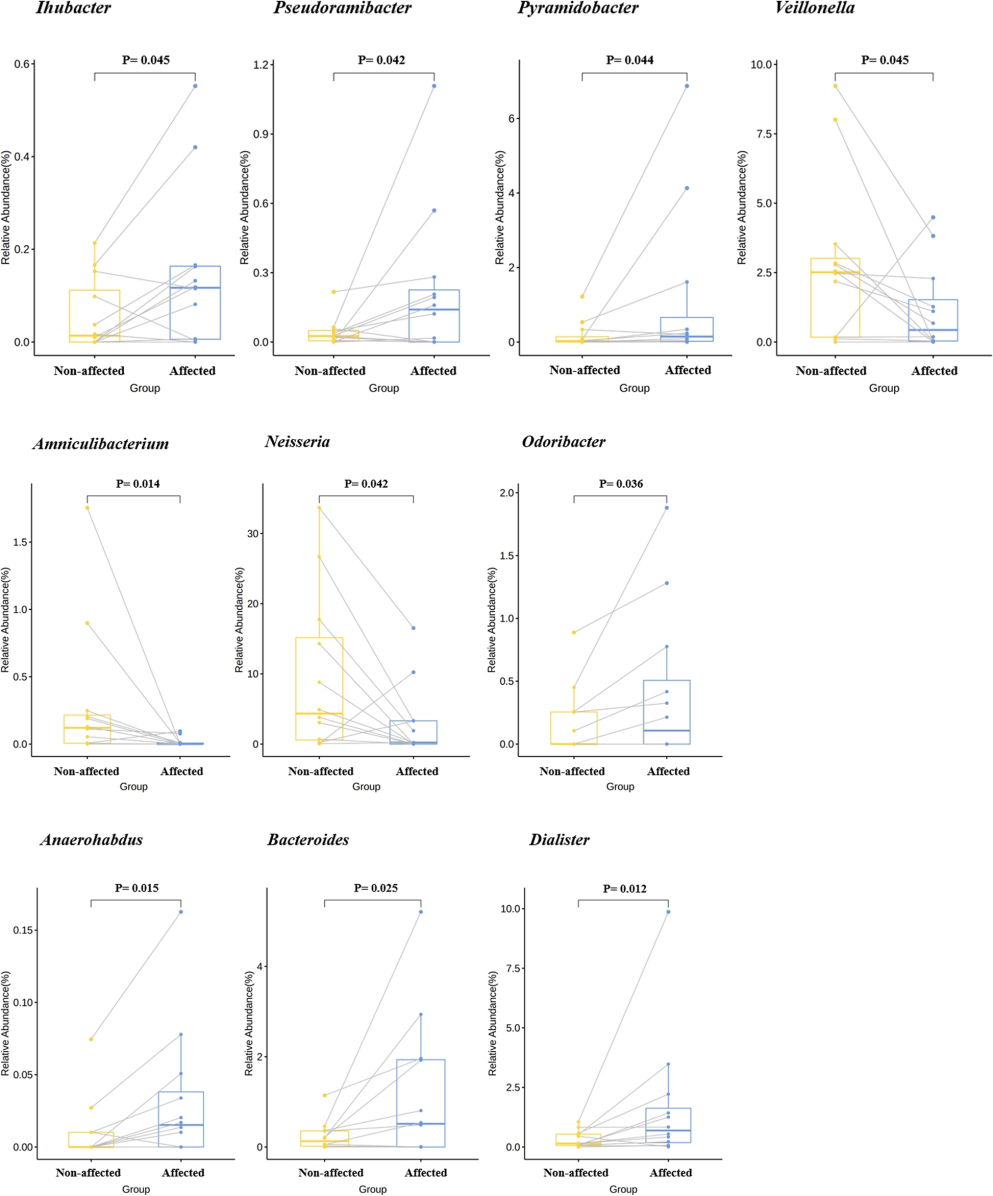
Oral microbiome comparison at the genus level between the two groups. Three genera reflect significantly higher relative abundances in the control group at the genus level. Seven genera are identified, reflecting significantly higher relative abundances in the experimental group

*Prevotella*, *Streptococcus*, *Porphyromonas*, and *Fusobacterium* were predominant in all samples. In addition, *Neisseria* and *Rothia* were detected at high levels in the unaffected group, but this was not statistically significant. There was a significant difference in the comparative analysis of relative abundance between the two groups. Three genera (*Amniculibacterium*, *Neisseria*, and *Veillonella*,*)* were identified in the unaffected group, reflecting a significantly higher relative abundance at the genus level. In the affected group, seven genera (*Anaerorhabdus*, *Bacteroides*, *Dialister*, *Ihubacter*, *Odoribacter*, *Pseudoramibacter, and pyramidobacter*) were identified, reflecting a significantly high relative abundance (Fig. 7).

#### Oral microbiome analysis at the species level

In total, 720 taxa were included in the species analysis. Of these, 23 taxa were identified to the species level. Taxa with an average abundance value of less than 1% in the entire sample were labelled as “other” (Supplementary Fig. 1).

*Streptococcus oralis*, *Porphyromonas gingivalis*, *Prevotella intermedia*, and *Fusobacterium nucleatum* were predominant in all samples. There was a significant difference in the comparative analysis of relative abundance between the two groups. Seven species with significantly high relative abundances in the unaffected group were identified at the species level. In contrast, eight species with significantly higher relative abundances were identified in the affected group (Supplementary Fig. 2).

## Discussion

MRONJ is rare condition, posing challenges in diagnosis and treatment selection. Since a variety of factors are reported to cause the same or similar symptoms as MRONJ in patients who have not been exposed to drugs known to cause MRONJ, it is challenging to prove a causal relationship [37–51]. Furthermore, many patients taking medications associated with MRONJ often present with other comorbidities that may aggravate, modify, or contribute to MRONJ. These confounding factors are complex and variable, making it difficult to determine the incidence and prevalence of MRONJ.

Recent hypotheses suggest infection as a potential cause of MRONJ, underscoring the importance of understanding the oral microbiome in identifying the etiology of MRONJ [17, 18, 29]. Traditional culture-based methods have limitations in exploring its etiology, which have led to an increased use of NGS in oral microbiome studies. Unlike conventional microbial culture tests, NGS is a culture-independent method that uses 16S rRNA gene sequencing. This technology has enabled the discovery of previously unidentified strains, allowing for more in-depth research. In this study, samples were collected from the affected and unaffected sides of patients with MRONJ to evaluate their characteristics through oral microbiome analysis.

Our study’s microbiome taxonomy results can be summarized as follows:

1. At the phylum level, the affected group showed a significantly lower presence of *Cyanobacteria,* while *Proteobacteria*, were lower and *Bacteroidetes* were more abundant, although difference was not statistically significant. *Bacteroidetes* are known to express exoenzymes and adhesion factors that can potentially delay wound healing or cause tissue damage, thereby contributing to early disease recurrence or impaired healing in conservative treatment approaches [52–54]. Additionally, one study reported an increase in *Bacteroidetes* with decreasing body weight in mice [55]. Considering these factors along with the current experimental results, targeting *Bacteroidetes* inhibition could potentially slow disease progression or assist in treatment. Additionally, providing sufficient nutritional support to patients to prevent weight loss during treatment may indirectly aid healing and prevent recurrence.
2. At the genus level, a combination of gram-positive and gram-negative bacteria were abundant. *Prevotella*, *Porphyromonas*, *Fusobacterium* and *Streptococcus* were predominant in all samples, with *Prevotella* and *Porphyromonas* showing a higher prevalence in the affected group, although the difference was not statistically significant. *Prevotella*, similar to *Bacteriodetes*, is known to potentially delay wound healing or cause tissue damage and is one of several species that can contribute to periodontitis development [52–54, 56]. Additionally, *Porphyromonas gingivalis* can cause high levels of bone resorption and associated destructive periodontitis [57, 58]. *Fusobacterium*, a gram-negative bacterium, forms colonies in supragingival and subgingival dental plaque and acts as a bridge connecting early and late microbial colonies in the pathogenesis of periodontal disease [59, 60]. In addition, streptococci are commonly found in periodontal disease and MRONJ lesions [61–65]; saccharolytic bacteria, including streptococci, create an acidic environment that can hinder wound healing [64]. Furthermore, *Streptococcus* is known to engage in intra- and inter-generic co-aggregation with other microorganisms such as *Actinomyces* spp., *Capnocytophaga* spp., *Eikenella* spp., *Prevotella* spp., and *Veillonella* spp. to form initial colonies [25]. Given the findings of this study, it is speculated that the oral cavity contains various microorganisms that can cause periodontitis, and their complex interactions, co-aggregation, and opportunistic infections can potentially influence the development and progression of MRONJ. Therefore, the regulation of these bacteria may be significant for MRONJ treatment. Indeed, regarding *Prevotella*, experiments have demonstrated that inhibiting this organism and activating *Lactobacillus* leads to increased production of inflammatory cytokines and a preventive effect against osteoradionecrosis [66].
3. In the affected group, *Synergistetes* was prominently present at the phylum level, *Pyramidobacter* was abundant at the genus level, and *Dialister pneumosinetes, Dialister ivisus*, and *Pseudoramibacter alatolyticus* were prevalent at the species level. These microorganisms are known to be associated with periapical infection and periodontal disease [26, 64, 67, 68]. Thus, the presence of these bacteria, which can cause periapical and periodontal infections, can potentially lead to MRONJ or exacerbate the condition in affected patients.
4. In this study, *Actinomyces* was lower in the affected group, although the difference was not statistically significant. Many studies have reported the presence of *Actinomyces*, which are known to adhere to the exposed bone in the oral cavity, form clusters, and create an anaerobic and acidic environment in nearby areas, thus interfering with the clustering of other bacterial groups and protecting against antibiotics and other local defense mechanisms [69]. *Actinomyces* spp. are associated with opportunistic infections; when they enter the submucosal space, they fight for survival [63, 70]. Therefore, the results may vary depending on the method or location of sample collection, the collection method, or the patient population. When analyzing the surrounding submucosal area to observe the microbiome, the abundance of *Actinomyces* may appear to be lower, as observed in our study. This finding aligns with Wei et al. (2012) [64], who reported a similar low abundance of *Actinomyces*. Conversely, Zirk et al. (2019) [69] found a low abundance of *Actinomyces* in bone samples but a higher abundance in the submucosal area [69]. To utilize this information clinically, standardization, including consistent sample collection methods and sites, is necessary.

Table 4 compares the findings of several previous studies, and clearly indicates that different microorganisms were identified in each study.

**Table 4:** Microorganisms reported in previous studies related to MRONJ

Authors, Date	Microorganisms
[64]	*Pseudoramibacter alactolyticus* and *Streptococcus mitis* were predominant in the BRONJ group (phyla). *Pseudoramibacter alactolyticus, S. mitis, Atopobium* sp. *Mogibacterium timidum*, and *Bacteroidetes bacterium* were predominant in the BRONJ group (species).
[25]	*Parvimonas micra, Streptococcus anginosus, Atopobium rimae, Peptostreptococcus stomatis* and *Eubacterium* were predominant in the BRONJ lesion.
[71]	*Streptococcus mutans, Staphylococcus aureus* and *Pseudomonas aeruginosa*
[72]	*Actinomyces* spp.*, Capnocytophaga* sp.*, Neisseria* sp., and other aerobes and anaerobes.
[61]	*Actinomyces* spp.
[69]	*Streptococcus* spp., *Prevotella* spp., and *Actinomyces* spp. in osteonecrosis bone and concomitant soft tissue and submucosal infection area.

*BRONJ* bisphosphonate-related osteonecrosis of jaw

However, the specific microorganisms that contribute to MRONJ remain unclear. Since Antony van Leeuwenhoek’s discovery of bacteria in the oral cavity in 1680, over 700 species of oral microorganisms, including bacteria, fungi, and viruses, have been identified in the teeth and oral mucosa [73, 74]. Even within a healthy oral cavity, a diverse range of microorganisms exist, with more than 200 of these 700 species remaining uncultured, highlighting the complexity of the oral ecosystem.

The oral microbiome shows considerable variation between individuals and within different regions of the same mouth. Various microbial strains are present on oral surfaces such as the tongue, cheeks, tonsils, and teeth; these microbes are constantly exposed to and adapt to changing physical conditions like temperature, humidity, masticatory force, and levels of nutrients and oxygen [32, 75, 76]. Furthermore, the microbial community at an infection site can greatly vary depending on the disease stage, leading to challenges in pinpointing the specific microorganisms that cause diseases such as MRONJ.

When selecting a control group, samples collected from a healthy person may lead to variables that cannot be controlled, such as patient’s general condition, environment, and diet [77–79]. To address this, our study employed a split-mouth design to elucidate the microbiome clustered around the lesion and control for some of the variables. This approach allowed us to compare the affected and unaffected sides in patients with MRONJ; using 16S rRNA metagenomics, we were able to identify a greater number of microorganisms comparing to using culture-based methods alone. However, it could not provide information about the presence, quantity, and function of specific taxa in the microbial community, resulting in discrepancies in microbial abundance and composition compared to previous studies.

Despite the limited sample size, our findings demonstrated the presence of diverse and unique bacterial communities in MRONJ, raising intriguing questions regarding the role of oral bacteria in MRONJ pathogenesis. However, it should be noted that the use of genetic amplification methods can introduce biases and potentially overestimate diversity, especially in cases where a few dominant species are present, which limits the ability to reveal the full extent of species diversity. Although we tried to avoid influencing the microbiome between groups as much as possible by using a split-mouth design, we still could not rule out an influence on our results, and the small sample size did not allow us to analyze the potential risk factors influencing MRONJ development or conduct comparative analyses based on MRONJ stages. Furthermore, our patient population was limited to Koreans in the Republic of Korea; therefore, regional and ethnic differences might have been present.

As a preliminary study, future studies should aim to include a larger number of samples for more robust results. Additionally, implementing propensity score matching with a healthy group will provide a more accurate comparison. Furthermore, we are considering collaboration with multiple institutions to facilitate a comparative analysis between different ethnic groups. These steps will help not only in addressing the limitations of the current study but also in expanding our understanding of the subject matter.

## Conclusions

The results obtained from the samples collected from the affected and unaffected sides of patients with MRONJ suggest that MRONJ is less likely to be attributed to a specific microorganism. Instead, it appears to be associated with factors disrupting oral homeostasis, such as periodontitis or periapical infections. These findings, when addressed in large-scale studies, can provide further insights into the role of the oral microbiome in the pathogenesis of MRONJ. This understanding may pave the way for the development of more effective therapeutic interventions for patients with MRONJ.

### Supplementary Information

**Additional file 1: Supplementary Table 1.** Taxonomic identification.

**Additional file 2: Supplementary Figure 1.** Oral microbiome analysis at the species level.

**Additional file 3: Supplementary Figure 2.** Oral microbiome comparison at the species level in both groups.

## Data Availability

No datasets were generated or analysed during the current study.

## Abbreviations

AAOMS: American association of oral and maxillofacial surgeons
ASV: Amplicon sequence variant
DADA2: Divisive amplicon denoising algorithm 2
DB: Database
DNA: Deoxyribonucleic acid
IRB: Institutional review board
MRONJ: Medication-related osteonecrosis of the jaw
NGS: Next-generation sequencing
PCR: Polymerase chain reaction
RNA: Ribonucleic acid

## References

[1] Khan AA, Morrison A, Hanley DA, Felsenberg D, McCauley LK, O'Ryan F, Reid IR, Ruggiero SL, Taguchi A, Tetradis S (2015). Diagnosis and management of osteonecrosis of the jaw: a systematic review and international consensus. J Bone Miner Res.

[2] Rosenberg TJ, Ruggiero S (2003). Osteonecrosis of the jaws associated with the use of bisphosphonates. J Oral Maxillofac Surg.

[3] Marx RE (2003). Pamidronate (Aredia) and zoledronate (Zometa) induced avascular necrosis of the jaws: a growing epidemic. J Oral Maxillofac Surg.

[4] Wang J, Goodger N, Pogrel M (2003). Osteonecrosis of the jaws associated with cancer chemotherapy1. J Oral Maxillofac Surg.

[5] Migliorati CA (2003). Bisphosphanates and oral cavity avascular bone necrosis. J Clin Oncol Off J Am Soc Clin Oncol.

[6] Fassio A, Bertoldo F, Idolazzi L, Viapiana O, Rossini M, Gatti D (2017). Drug-induced osteonecrosis of the jaw: the state of the art. Reumatismo.

[7] Fedele S, Bedogni G, Scoletta M, Favia G, Colella G, Agrillo A, Bettini G, Di Fede O, Oteri G, Fusco V (2015). Up to a quarter of patients with osteonecrosis of the jaw associated with antiresorptive agents remain undiagnosed. Br J Oral Maxillofac Surg.

[8] Schiodt M, Reibel J, Oturai P, Kofod T (2014). Comparison of nonexposed and exposed bisphosphonate-induced osteonecrosis of the jaws: a retrospective analysis from the Copenhagen cohort and a proposal for an updated classification system. Oral Surg Oral Med Oral Pathol Oral Radiol.

[9] Patel S, Choyee S, Uyanne J, Nguyen A, Lee P, Sedghizadeh P, Kumar S, Lytle J, Shi S, Le A (2012). Non-exposed bisphosphonate-related osteonecrosis of the jaw: a critical assessment of current definition, staging, and treatment guidelines. Oral Dis.

[10] Chan BH, Yee R, Puvanendran R, Ang SB (2018). Medication-related osteonecrosis of the jaw in osteoporotic patients: prevention and management. Singap Med J.

[11] Shoback D (2007). Update in osteoporosis and metabolic bone disorders. J Clin Endocrinol Metabol..

[12] Assaf AT, Smeets R, Riecke B, Weise E, Groebe A, Blessmann M, Steiner T, Wikner J, Friedrich RE, Heiland M (2013). Incidence of bisphosphonate-related osteonecrosis of the jaw in consideration of primary diseases and concomitant therapies. Anticancer Res.

[13] Ruggiero SL, Mehrotra B (2009). Bisphosphonate-related osteonecrosis of the jaw: diagnosis, prevention, and management. Annu Rev Med.

[14] Galis B, Zajko J, Hirjak D, Vanko L, Kupcova I, Jurkemik J, Gengelova P, Mikuskova K, Halmova K, Riznic M (2017). Is the prevalence of the medication-related osteonecrosis of the jaws underestimated, evaluation in oncological and non-oncological disease. Bratisl Lek Listy..

[15] Ulmner M, Jarnbring F, Törring O (2014). Osteonecrosis of the jaw in Sweden associated with the oral use of bisphosphonate. J Oral Maxillofac Surg.

[16] Coello-Suanzes J, Rollon-Ugalde V, Castaño-Seiquer A, Lledo-Villar E, Herce-Lopez J, Infante-Cossio P, Rollon-Mayordomo A (2018). Preventive dental management of osteonecrosis of the jaws related to zoledronic acid treatment. Oral Dis.

[17] Boff RC, Salum FG, Figueiredo MA, Cherubini K (2014). Important aspects regarding the role of microorganisms in bisphosphonate-related osteonecrosis of the jaws. Arch Oral Biol.

[18] Cerrato A, Zanette G, Boccuto M, Angelini A, Valente M, Bacci C (2021). Actinomyces and MRONJ: a retrospective study and a literature review. J Stomatol Oral Maxillofac Surg..

[19] De Ceulaer J, Tacconelli E, Vandecasteele S (2014). Actinomyces osteomyelitis in bisphosphonate-related osteonecrosis of the jaw (BRONJ): the missing link?. Eur J Clin Microbiol Infect Dis.

[20] Naik NH, Russo TA (2009). Bisphosphonate-related osteonecrosis of the jaw: the role of actinomyces. Clin Infect Dis.

[21] Otto S, Hafner S, Mast G, Tischer T, Volkmer E, Schieker M, Stürzenbaum SR, von Tresckow E, Kolk A, Ehrenfeld M (2010). Bisphosphonate-related osteonecrosis of the jaw: is pH the missing part in the pathogenesis puzzle?. J Oral Maxillofac Surg.

[22] Song M, Alshaikh A, Kim T, Kim S, Dang M, Mehrazarin S, Shin K-H, Kang M, Park N-H, Kim RH (2016). Preexisting periapical inflammatory condition exacerbates tooth extraction–induced bisphosphonate-related osteonecrosis of the jaw lesions in mice. J Endod.

[23] Schipmann S, Metzler P, Rössle M, Zemann W, von Jackowski J, Obwegeser J, Grätz K, Jacobsen C (2013). Osteopathology associated with bone resorption inhibitors–which role does Actinomyces play? A presentation of 51 cases with systematic review of the literature. J Oral Pathol Med..

[24] Kumar SK, Gorur A, Schaudinn C, Shuler CF, Costerton JW, Sedghizadeh PP (2010). The role of microbial biofilms in osteonecrosis of the jaw associated with bisphosphonate therapy. Curr Osteoporos Rep..

[25] Pushalkar S, Li X, Kurago Z, Ramanathapuram LV, Matsumura S, Fleisher KE, Glickman R, Yan W, Li Y, Saxena D (2014). Oral microbiota and host innate immune response in bisphosphonate-related osteonecrosis of the jaw. Int J Oral Sci..

[26] Sedghizadeh PP, Kumar SK, Gorur A, Schaudinn C, Shuler CF, Costerton JW (2008). Identification of microbial biofilms in osteonecrosis of the jaws secondary to bisphosphonate therapy. J Oral Maxillofac Surg.

[27] Almazrooa SA, Woo S-B (2009). Bisphosphonate and nonbisphosphonate-associated osteonecrosis of the jaw: a review. J Am Dent Assoc.

[28] Kalyan S, Wang J, Quabius ES, Huck J, Wiltfang J, Baines JF, Kabelitz D (2015). Systemic immunity shapes the oral microbiome and susceptibility to bisphosphonate-associated osteonecrosis of the jaw. J Transl Med.

[29] De Bruyn L, Coropciuc R, Coucke W, Politis C (2018). Microbial population changes in patients with medication-related osteonecrosis of the jaw treated with systemic antibiotics. Oral Surg Oral Med Oral Pathol Oral Radiol.

[30] Peterson J, Garges S, Giovanni M, McInnes P, Wang L, Schloss JA, Bonazzi V, McEwen JE, Wetterstrand KA, Deal C (2009). The NIH human microbiome project. Genome Res.

[31] Do T, Devine D, Marsh PD. Oral biofilms: molecular analysis, challenges, and future prospects in dental diagnostics. Clin Cosmet Investig Dent. 2013:11–9. 10.2147/CCIDE.S31005 PMC3652372 23674928

[32] The Human Microbiome Project Consortium (2012). Structure, function and diversity of the healthy human microbiome. Nature..

[33] Wade WG (2013). The oral microbiome in health and disease. Pharmacol Res.

[34] Dawes C (1987). Physiological factors affecting salivary flow rate, oral sugar clearance, and the sensation of dry mouth in man. J Dent Res.

[35] Integrative HMP (iHMP) Research Network Consortium. The integrative human microbiome project. Nature. 2019;569:641–8. 10.1038/s41586-019-1238-8 PMC6784865 31142853

[36] Bolyen E, Rideout JR, Dillon MR, Bokulich NA, Abnet CC, Al-Ghalith GA, Alexander H, Alm EJ, Arumugam M, Asnicar F (2019). Reproducible, interactive, scalable and extensible microbiome data science using QIIME 2. Nat Biotechnol.

[37] Farah C, Savage N (2003). Oral ulceration with bone sequestration. Aust Dent J.

[38] Filippi A, Dreyer T, Bohle RM, Pohl Y, Rosseau S (1997). Sequestration of the alveolar bone by invasive aspergillosis in acute myeloid leukemia. J Oral Pathol Med..

[39] Friel P, Macintyre D (2002). Bone sequestration from lower 3rd molar region. Br Dent J.

[40] Huang J-S, Kok S-H, Lee J-J, Hsu W-Y, Chiang C-P, Kuo Y-S (2005). Extensive maxillary sequestration resulting from mucormycosis. Br J Oral Maxillofac Surg.

[41] Peters E, Daley T (2003). Persistent painful ulcer of the posterior lingual mandibular mucosa. J Contemp Dent Pract.

[42] Sonnier KE, Horning GM (1997). Spontaneous bony exposure: a report of 4 cases of idiopathic exposure and sequestration of alveolar bone. J Periodontol.

[43] Peters E, Lovas G, Wysocki G (1993). Lingual mandibular sequestration and ulceration. Oral Surg Oral Med Oral Pathol..

[44] Nandakumar H, Shankaramba K (1990). Massive sequestration of the upper jaw: a case report. Br J Oral Maxillofac Surg.

[45] Ramon Y, Oberman M, Horowitz I, Freedman A (1977). Extensive maxillary sequestration resulting from rhinocerebral mucormyocosis. J Oral Surg (American Dental Association: 1965)..

[46] Liao M-T, Chien W-C, Wang J-C, Chung C-H, Chu S-J, Tsai S-H (2019). Increased risk of bisphosphonate-related osteonecrosis of the jaw in patients with Sjögren’s syndrome: nationwide population-based cohort study. BMJ Open.

[47] Schwartz HC (1982). Osteonecrosis of the jaws: a complication of cancer chemotherapy. Head Neck Surg..

[48] Cooper J (1977). Tooth exfoliation and osteonecrosis of the jaw following herpes zoster. Br Dent J.

[49] Schwartz O, Kvorning S (1982). Tooth exfoliation, osteonecrosis of the jaw and neuralgia following herpes zoster of the trigeminal nerve. Int J Oral Surg.

[50] Calhoun KH, Shapiro RD, Stiernberg CM, Calhoun JH, Mader JT (1988). Osteomyelitis of the mandible. Arch Otolaryngol–Head Neck Surg..

[51] Koorbusch GF, Fotos P, Goll KT (1992). Retrospective assessment of osteomyelitis: etiology, demographics, risk factors, and management in 35 cases. Oral Surg Oral Med Oral Pathol..

[52] Bowler P, Duerden B, Armstrong DG (2001). Wound microbiology and associated approaches to wound management. Clin Microbiol Rev.

[53] Stefanopoulos PK, Kolokotronis AE (2004). The clinical significance of anaerobic bacteria in acute orofacial odontogenic infections. Oral Surg Oral Med Oral Pathol Oral Radiol Endodontol..

[54] Duerden BI (1994). Virulence factors in anaerobes. Clin Infect Dis.

[55] Cao TT, Wu K-C, Hsu J-L, Chang C-S, Chou C, Lin C-Y, Liao Y-M, Lin P-C, Yang L-Y, Lin H-W (2020). Effects of non-insulin anti-hyperglycemic agents on gut microbiota: a systematic review on human and animal studies. Front Endocrinol.

[56] Ibrahim M, Subramanian A, Anishetty S (2017). Comparative pan genome analysis of oral Prevotella species implicated in periodontitis. Funct Integr Genomics.

[57] Kesavalu L, Bakthavatchalu V, Rahman M, Su J, Raghu B, Dawson D, Fernandes G, Ebersole J (2007). Omega-3 fatty acid regulates inflammatory cytokine/mediator messenger RNA expression in Porphyromonas gingivalis-induced experimental periodontal disease. Oral Microbiol Immunol.

[58] Kesavalu L, Sathishkumar S, Bakthavatchalu V, Matthews C, Dawson D, Steffen M, Ebersole JL (2007). Rat model of polymicrobial infection, immunity, and alveolar bone resorption in periodontal disease. Infect Immun.

[59] Kolenbrander PE, London J (1993). Adhere today, here tomorrow: oral bacterial adherence. J Bacteriol.

[60] Socransky SS, Haffajee AD (2005). Periodontal microbial ecology. Periodontology.

[61] Panya S, Fliefel R, Probst F, Tröltzsch M, Ehrenfeld M, Schubert S, Otto S (2017). Role of microbiological culture and polymerase chain reaction (PCR) of actinomyces in medication-related osteonecrosis of the jaw (MRONJ). J Cranio-Maxillofac Surg.

[62] Kos M, Luczak K (2009). Bisphosphonates promote jaw osteonecrosis through facilitating bacterial colonisation. Biosci Hypotheses..

[63] Hinson A, Smith C, Siegel E, Stack B. Is bisphosphonate-related osteonecrosis of the jaw an infection? A histological and microbiological ten-year summary. Int J Dent. 2014;2014. 10.1155/2014/452737 PMC4095654 25089126

[64] Wei X, Pushalkar S, Estilo C, Wong C, Farooki A, Fornier M, Bohle G, Huryn J, Li Y, Doty S (2012). Molecular profiling of oral microbiota in jawbone samples of bisphosphonate-related osteonecrosis of the jaw. Oral Dis.

[65] Hallmer F, Bjørnland T, Andersson G, Becktor JP, Kristoffersen AK, Enersen M (2017). Bacterial diversity in medication-related osteonecrosis of the jaw. Oral Surg Oral Med Oral Pathol Oral Radiol.

[66] Lukens JR, Gurung P, Vogel P, Johnson GR, Carter RA, McGoldrick DJ, Bandi SR, Calabrese CR, Walle LV, Lamkanfi M (2014). Dietary modulation of the microbiome affects autoinflammatory disease. Nature.

[67] Lee Y-H, Park HJ, Jeong S-J, Auh Q-S, Jung J, Lee G-J, et al. Oral microbiome profile of gingivitis and periodontitis by next-generation sequencing. 2023. 10.21203/rs.3.rs-3530768/v1. 10.1016/j.job.2024.100591 39581260

[68] Badros AZ, Meddeb M, Weikel D, Philip S, Milliron T, Lapidus R, Hester L, Goloubeva O, Meiller TF, Mongodin EF (2021). Prospective observational study of bisphosphonate-related osteonecrosis of the jaw in multiple myeloma: microbiota profiling and cytokine expression. Front Oncol.

[69] Zirk M, Wenzel C, Buller J, Zöller JE, Zinser M, Peters F (2019). Microbial diversity in infections of patients with medication-related osteonecrosis of the jaw. Clin Oral Investig.

[70] Thukral R, Shrivastav K, Mathur V, Barodiya A, Shrivastav S (2017). Actinomyces: a deceptive infection of oral cavity. J Korean Assoc Oral Maxillofac Surg.

[71] Kos M, Junka A, Smutnicka D, Szymczyk P, Gluza K, Bartoszewicz M (2015). Bisphosphonates enhance bacterial adhesion and biofilm formation on bone hydroxyapatite. J Cranio-Maxillofac Surg.

[72] Real CV, Sayáns MP, Suárez-Peñaranda J, Sanchez FP, Vila PG, Carrión AB (2016). Role of microbiota and inflammation in osteonecrosis of the jaw. Int J Clin Exp Pathol.

[73] Peters BA, Wu J, Pei Z, Yang L, Purdue MP, Freedman ND, Jacobs EJ, Gapstur SM, Hayes RB, Ahn J (2017). Oral microbiome composition reflects prospective risk for esophageal cancers. Cancer Res.

[74] Dewhirst FE, Chen T, Izard J, Paster BJ, Tanner AC, Yu W-H, Lakshmanan A, Wade WG (2010). The human oral microbiome. J Bacteriol.

[75] Hall MW, Singh N, Ng KF, Lam DK, Goldberg MB, Tenenbaum HC, JDG N, Beiko R, Senadheera DB (2017). Inter-personal diversity and temporal dynamics of dental, tongue, and salivary microbiota in the healthy oral cavity. NPJ Biofilms Microbiomes.

[76] Keijser B, Zaura E, Huse S, Van Der Vossen J, Schuren F, Montijn R, Ten Cate J, Crielaard W (2008). Pyrosequencing analysis of the oral microflora of healthy adults. J Dent Res.

[77] Costello EK, Lauber CL, Hamady M, Fierer N, Gordon JI, Knight R (2009). Bacterial community variation in human body habitats across space and time. Science.

[78] Nasidze I, Li J, Quinque D, Tang K, Stoneking M (2009). Global diversity in the human salivary microbiome. Genome Res.

[79] Wu Y, Chi X, Zhang Q, Chen F, Deng X (2018). Characterization of the salivary microbiome in people with obesity. PeerJ.

