# Supplementary material for: Can medication-related osteonecrosis of the jaw be attributed to specific microorganisms through oral microbiota analyses? A preliminary study
Source: BMC Oral Health. 2024 Feb 1;24:160. doi: 10.1186/s12903-024-03945-z (PMC10832156; doi:10.1186/s12903-024-03945-z)
Supplement: Supplementary file 1 — Additional file 1: Supplementary Table 1. Taxonomic identification. [file 12903_2024_3945_MOESM1_ESM.docx]

Supplementary Table 1. Taxonomic identification

| **Affected Group** | |  |  | **Unaffected Group** | |  |  |
| --- | --- | --- | --- | --- | --- | --- | --- |
| **Patient** | **Raw**  **Reads** | **Final**  **Reads** | **ASVs** | **Patient** | **Raw**  **Reads** | **Final**  **Reads** | **ASVs** |
| **1** | 91,797 | 74,681 | 197 | **1** | 105,934 | 87,377 | 159 |
| **2** | 98,932 | 82,350 | 127 | **2** | 106,312 | 88,992 | 288 |
| **3** | 56,192 | 29,510 | 123 | **3** | 79,320 | 61,569 | 164 |
| **4** | 65,311 | 57,873 | 85 | **4** | 62,956 | 53,274 | 99 |
| **5** | 89,258 | 71,625 | 148 | **5** | 88,289 | 72,978 | 261 |
| **6** | 101,280 | 66,968 | 134 | **6** | 75,748 | 61,469 | 132 |
| **7** | 132,252 | 85,828 | 121 | **7** | 153,731 | 97,604 | 344 |
| **8** | 75,652 | 44,840 | 170 | **8** | 82,695 | 53,920 | 549 |
| **9** | 94,706 | 44,483 | 213 | **9** | 82,716 | 47,568 | 172 |
| **10** | 97,887 | 61,980 | 234 | **10** | 98,475 | 61,264 | 231 |
| **11** | 92,360 | 50,626 | 265 | **11** | 106,820 | 58,623 | 300 |
| **12** | 107,258 | 51,180 | 169 | **12** | 77,423 | 39,684 | 147 |
| **Average** | **91,907** | **60,162** | **166** | **Average** | **93,368** | **65,360** | **237** |

ASV, amplicon sequence variants.
