# Supplementary figures and images for: Can medication-related osteonecrosis of the jaw be attributed to specific microorganisms through oral microbiota analyses? A preliminary study
Source: BMC Oral Health. 2024 Feb 1;24:160. doi: 10.1186/s12903-024-03945-z (PMC10832156; doi:10.1186/s12903-024-03945-z)

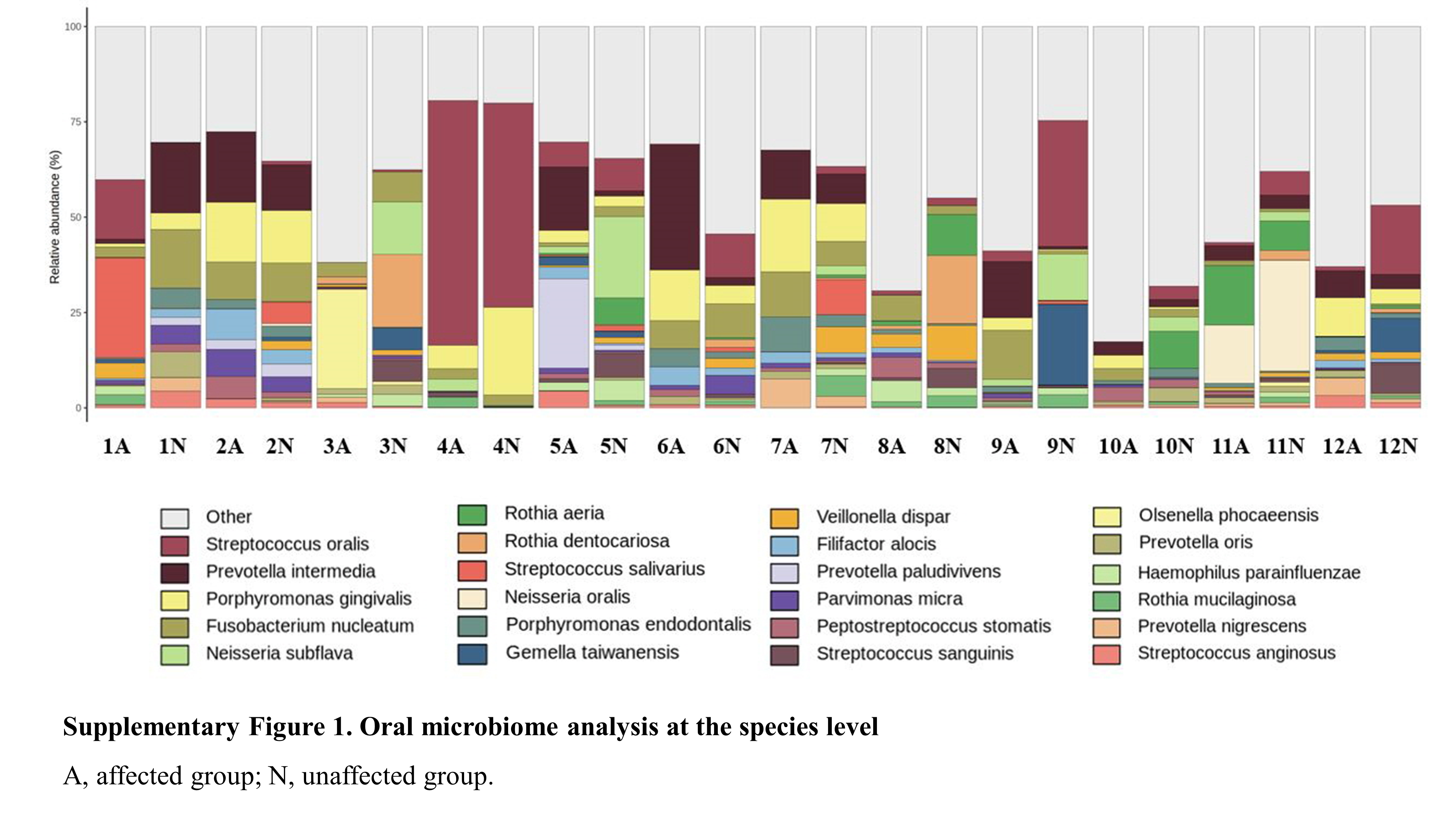

Supplement: Supplementary file 2 — Additional file 2: Supplementary Figure 1. Oral microbiome analysis at the species level. [file 12903_2024_3945_MOESM2_ESM.tif]

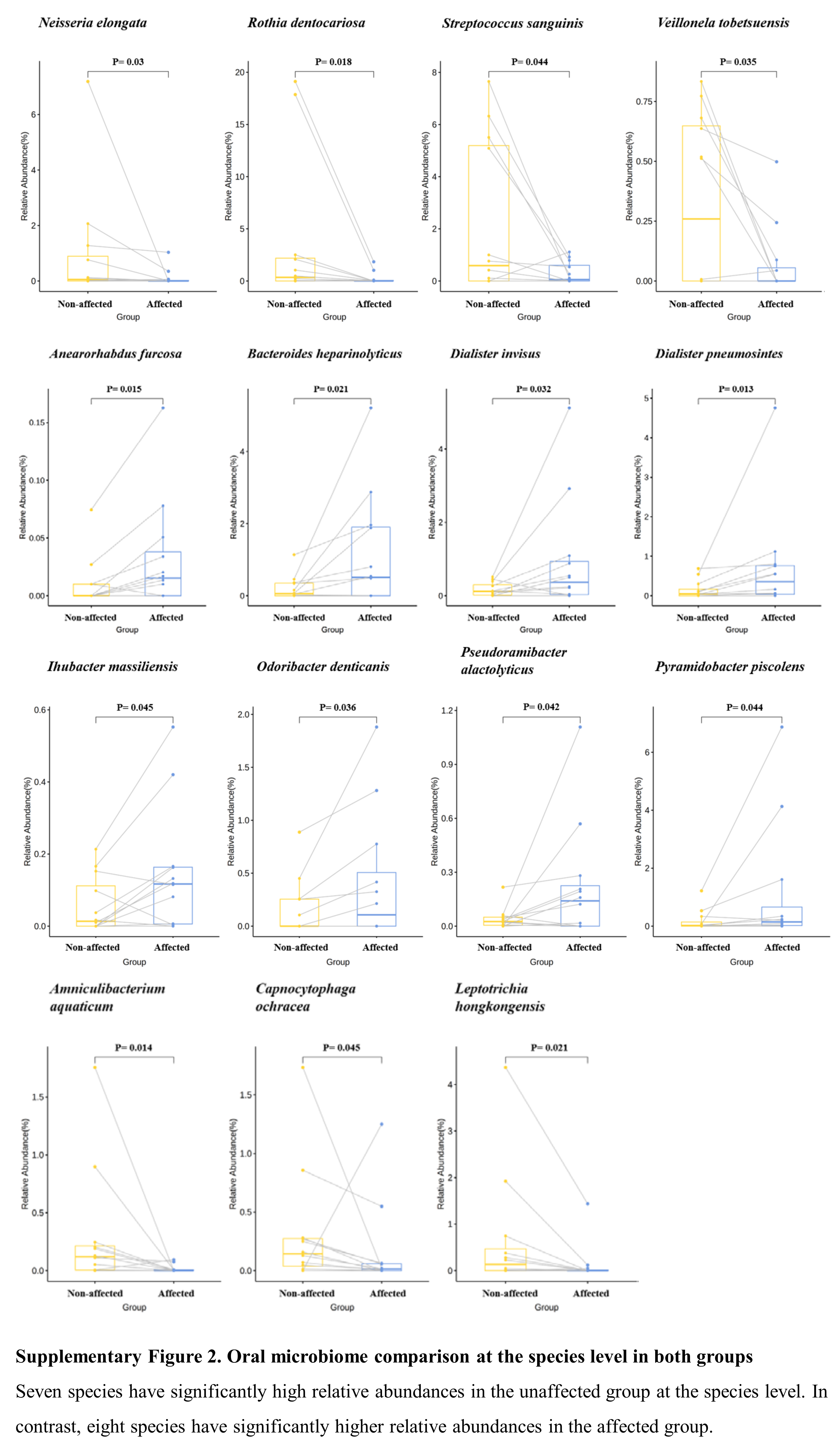

Supplement: Supplementary file 3 — Additional file 3: Supplementary Figure 2. Oral microbiome comparison at the species level in both groups. [file 12903_2024_3945_MOESM3_ESM.tif]
